# Substance P selectively decreases paired pulse depression in the rat hippocampal slice

**DOI:** 10.1186/1471-2202-6-66

**Published:** 2005-11-23

**Authors:** Kerrie N Wease, Stephen N Davies

**Affiliations:** 1 Department of Biomedical Sciences, Institute of Medical Sciences, University of Aberdeen, Foresterhill, Aberdeen, AB25 2ZD, Scotland, UK

## Abstract

**Background:**

Although being widespread in the hippocampus, the role tachykinins play in synaptic transmission is unclear. The effect of substance P on field potentials evoked by stimulation of the Schaffer collateral-commissural fibres and recorded from the CA1 region of the rat hippocampal slice were studied.

**Results:**

Perfusion of substance P (8 μM) had no effect on the fEPSP or population spike. Substance P did however cause a selective reduction in the paired pulse depression of population spikes evoked by paired stimulation at interpulse intervals of 20–80 msec. A comparison of the actions of other tachykinin receptor agonists gave an order of potency of substance P > [β-Ala^8^]-neurokinin A (4–10) > senktide. The effect of substance P was reduced by the neurokinin-1 receptor antagonist SR140333, but not by the neurokinin-2 or neurokinin-3 receptor antagonists, MDL 29,913 or [Trp^7^, β-Ala^8^]-neurokinin A (4–10).

**Conclusion:**

The order of potency of the agonists, and the effects of the antagonists, both indicate that the effect of substance P on paired pulse depression is mediated by neurokinin-1 receptors.

## Background

The mammalian tachykinins are a group of peptides sharing the common C-terminal sequence Phe-X-Gly-Leu-Met-NH_2_. The three principal tachykinins are substance P, neurokinin A and neurokinin B, and although these are preferred agonists for the neurokinin-1, neurokinin-2 and neurokinin-3 receptors respectively, they are not completely selective for any one receptor subtype [1,2]. Tachykinin receptors are distributed throughout the CNS, with all three receptor subtypes being expressed in the adult rat hippocampus [3-6]. A dense network of fibres containing substance P innervates the *stratum oriens*, *stratum radiatum *and *alveus *of the rat hippocampus. These may arise from both extrinsic sources such as the septum and hypothalamus, and from intrinsic GABA-containing interneurones [7,8].

Although being widespread in the hippocampus, the role tachykinins play in normal synaptic transmission is unclear. Using extracellular recordings from the mouse hippocampal slice, substance P and its analogue substance P methyl ester have been reported to cause a decrease in the amplitude and slope of the field excitatory postsynaptic potential (fEPSP) recorded from the CA1 *stratum pyramidale *[9]. The effect was blocked by the selective neurokinin-1 receptor antagonist SR140333, suggesting the action was NK-1 receptor mediated. The effect of substance P methyl ester was blocked by bicuculline, an antagonist for GABA_A _receptors, and not by glutamate receptor antagonists. The authors concluded the depressant effect of substance P and substance P methyl ester required an intact GABAergic system, with substance P causing facilitation of GABAergic neurotransmission, thereby increasing inhibitory synaptic transmission [9].

The aim of this present study was to use extracellular field recordings to a) identify the effect of substance P on synaptic transmission in the CA1 region of the rat hippocampus, and b) to use selective pharmacological agonists and antagonists to determine which tachykinin receptors were involved.

## Results

### Substance P had no effect on fEPSP's

**Figure 1:**
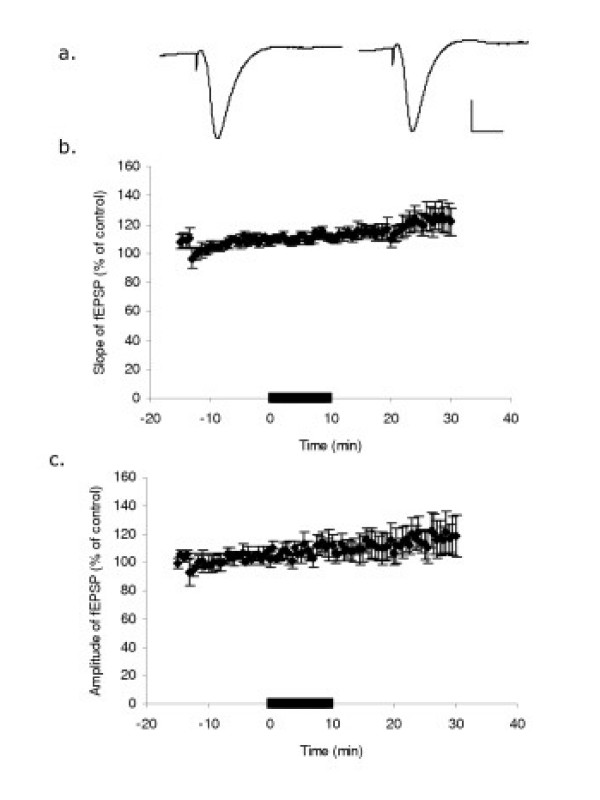
Perfusion of substance P (15 μM) had no effect on the amplitude or slope of the fEPSP recorded from the CA1 region of the rat hippocampal slice. (a) Example synaptic response recorded from a single slice using a single stimulus of the Schaffer collateral-commissural fibres every 30 s. The response on the left was recorded under control conditions whereas the trace on the right was recorded in the presence of substance P (15 μM). (b) and (c) Pooled time course data showing the lack of effect of substance P on the slope (b) and amplitude (c) of the fEPSP. Points represent mean ± s.e.m., n = 4. Scale bar represents 0.5 mV and 10 ms.

fEPSPs were recorded from the CA1 region of the rat hippocampus using single pulse stimulation of the Schaffer collateral commissural fibres at 30 s intervals. Perfusion of 15 μM substance P for 10 min had no significant effect on the amplitude of the fEPSP (106 ± 5% of control at the end of drug perfusion, figure 1(a) and 1(b), not significant) or the slope of the fEPSP (113 ± 2% of control at the end of drug perfusion, figure 1(a) and 1(c), not significant).

Contrary to previous experiments performed in the mouse hippocampus [9]), we therefore found no effect of substance P on fEPSPs recorded in the rat hippocampus. Existing immunohistochemical and electrophysiological data point to the fact that substance P receptors are found solely on inhibitory interneurones in the hippocampus [8,10]. In our recording conditions, GABAergic transmission plays a minimal role in determining the slope or amplitude of the fEPSP. We therefore turned to recording synaptic responses in which GABAergic transmission clearly has an effect. Synaptic stimulation of CA1 pyramidal neurones evokes a powerful feedback inhibition, which is mediated by GABA_A _receptors [11]. Paired pulse stimulation can be used to evoke a second response during this phase inhibition and the extent of paired pulse depression can be used as an index of the strength of GABAergic transmission [12]. We therefore investigated next the action of substance P on paired pulse depression of population spikes.

### Substance P decreased paired pulse depression

**Figure 2:**
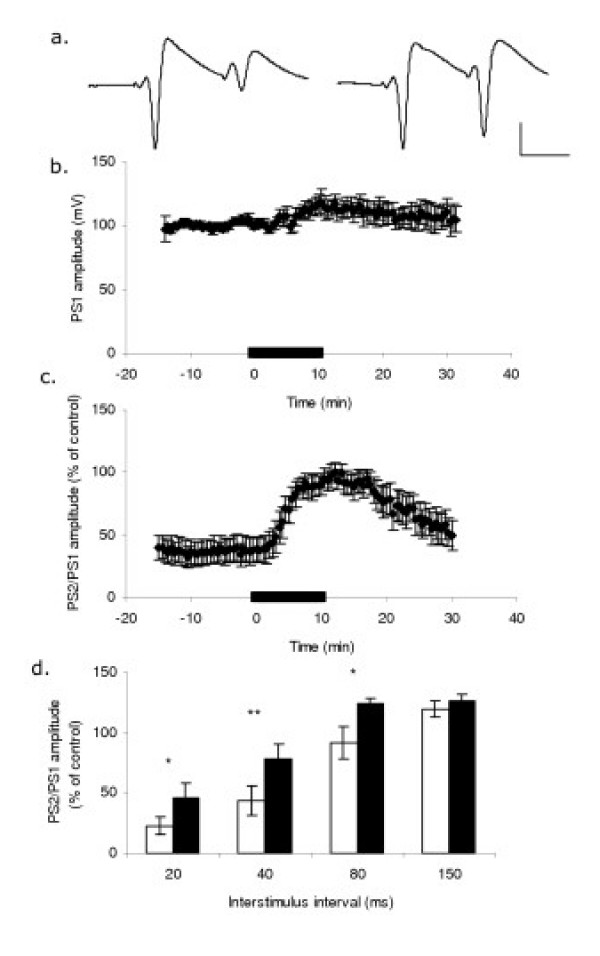
Perfusion of substance P (8 μM) had no effect on the amplitude of PS1 but reduced paired pulse depression. (a) Example synaptic response recorded from a single slice using paired pulse stimulation at an interstimulus interval of 20 ms. The response on the left was recorded under control conditions whereas the trace on the right is that recorded in the presence of substance P (8 μM). (b) Pooled time course data showing that perfusion of substance P for 10 min (black bar) had no effect on the amplitude of PS1. (c) Pooled time course data from the same set of experiments showing PS2 expressed as a % of PS1. Substance P (8 μM) perfused for 10 min (black bar) significantly increased PS2 amplitude and therefore decreased paired pulse depression. Points represent mean ± s.e.m., n = 7. (d) The effect of substance P on the full range of interstimulus intervals recorded during control conditions (open bars) and during drug perfusion (filled bars). * denotes p < 0.05, ** denotes p > 0.01, n = 9. Scale bar represents 1 mV and 10 ms.

There is some variability in the extent of paired pulse depression observed between individual slices. In general, those slices deemed healthy by the criterion of not showing a secondary population spike to a single stimulus, also show good paired pulse depression. Overall, ie prior to any selection of slices, when pulses were delivered 20 ms apart, the mean amplitude of the second population spike (PS2) was 55 ± 11% of the amplitude of the first (PS1) (n = 10). For the following experiments however, unless otherwise stated, only those slices in which PS2 was 70% or less of PS1 when using a 20 ms interpulse interval were used. Slices were stimulated every 30 s with paired pulses delivered 20 ms apart. A control period of at least 15 min was established, in which both PS1 amplitude and extent of paired pulse depression were stable, and then substance P was applied. Perfusion of 8 μM substance P for 10 minutes was found to cause an increase in the amplitude of PS2 while having no effect on PS1 (figure 2(b) and 2(c), n = 7). The mean amplitude of PS2 during the control period was 37 ± 9% of PS1, and this increased to 88 ± 8% of PS1 (p > 0.05) at the end of drug perfusion. This increase in PS2 was not accompanied by any significant effect on the amplitude of PS1, which was 114 ± 8% of control at the end of drug perfusion (p = 0.1 not significant). The peak effect of substance P on paired pulse depression was observed within 10 min of perfusion, and the effect was reversible towards control levels over 20–30 min.

In a different set of experiments, we next examined whether the effect of substance P was limited to those interpulse intervals that correspond with the time course of GABAergic feedback inhibition. Using paired pulse stimulation at interpulse intervals of 20, 40, 80 and 150 ms, the effect of 8 μM substance P perfused for 10 min was studied. The effect of substance P was evident only at the shorter interpulse intervals of 20–80 ms (figure 2(d), n = 9). The greatest increase in amplitude of PS2 was observed at the 40 ms interpulse interval where the control amplitude of PS2 was 43 ± 12% of PS1, but increased to 78 ± 12% of PS1 at the end of drug perfusion (p = 0.005). Substance P also caused a smaller but still significant increase in the amplitude of PS2 at the 20 ms interpulse interval (PS2 amplitude increased from 23 ± 7 to 45 ± 13% of PS1, p = 0.03) and the 80 ms interpulse interval (PS2 amplitude increased from 92 ± 14 to 124 ± 4% of PS1, p = 0.03). In contrast, substance P had no significant effect at the 150 ms interpulse interval (p = 0.2). Further to this, in the minority of slices which, even at short interpulse intervals showed no paired pulse depression, perfusion of 8 μM substance P had no effect on the amplitude of the first or second population spike (n = 3, data not shown).

### Effect of other tachykinin agonists on paired pulse depression

Substance P is most potent at the neurokinin-1 receptor, however, it can also activate the other tachykinin receptors (neurokinin-2 and neurokinin-3). Establishing an order of potency of selective agonists in mimicking the effect of substance P is therefore a useful indicator of which receptor mediates the effect.

**Figure 3:**
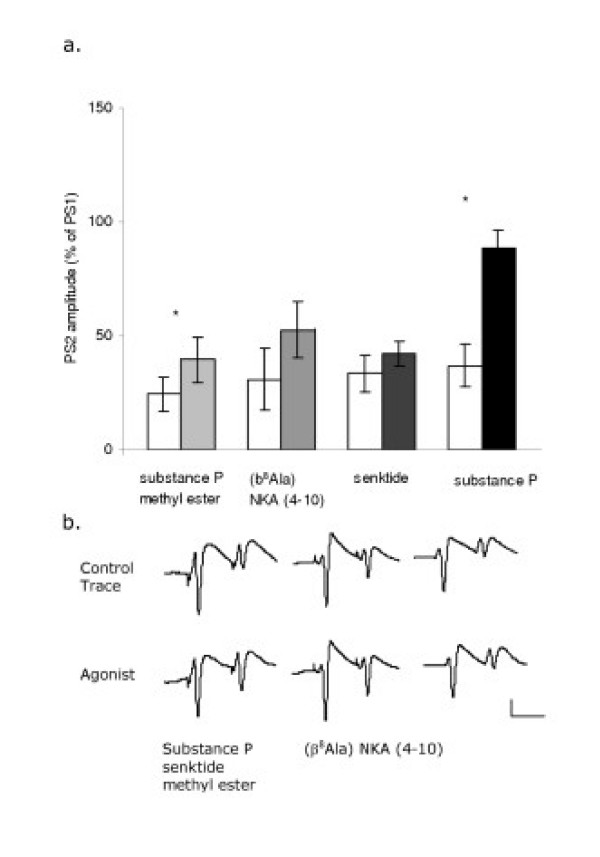
The effect of tachykinin receptor agonists on paired pulse depression. All agonists were perfused for 10 min. (a) Bars represents the control paired pulse depression using an interstimulus interval of 20 ms (PS2 amplitude expressed as a % of PS1, open bars), compared to that recorded in the presence of each agonist (filled bars). Substance P methyl ester significantly increased the amplitude of PS2, p > 0.05, n = 9. (β^8^Ala) neurokinin A (4–10) and senktide failed to have any significant effect on the amplitude of PS2, n = 4 for both. Substance P significantly increased the amplitude of PS2, p > 0.05, n = 7. (b) Example synaptic response recorded from 3 different representative slices. Top row of traces are controls, bottom traces are those recorded during perfusion of each tachykinin agonist. Scale bar represents 1 mV and 10 ms.

Substance P methyl ester has been reported to be a potent neurokinin-1 receptor agonist [13]. Using paired pulse stimulation with an interpulse interval of 20 ms, perfusion of 0.5 μM substance P methyl ester for 10 min had no effect on PS1, but significantly increased the amplitude of PS2, though the effect appeared smaller than that produced by 8 μM substance P. Substance P methyl ester produced an increase in PS2 amplitude from 21 ± 7% to 39 ± 10% of PS1 at the end of drug perfusion (p < 0.05, figure 3, n = 8), while having no effect on PS1 (PS1 amplitude was 114 ± 9% of control at the end of drug perfusion).

[β^8^Ala]-neurokinin A (4–10) is an neurokinin-2 receptor preferring agonist [14]. Using paired pulse stimulation with an interpulse interval of 20 ms, perfusion of 10 μM [β^8^Ala]-neurokinin A (4–10) for 10 min had a small and statistically insignificant effect on PS2. PS2 amplitude increased from 31 ± 13% to 51 ± 13% of PS1 at the end of drug perfusion (not significant, figure 3, n = 4). It too had no effect on PS1 (amplitude of PS1 was 99 ± 9% of control at the end of drug perfusion). In order to directly compare the effect of substance P in the same slices, after a 30-min washout, 8 μM substance P was perfused for 10 min. This increased the amplitude of PS2 from 34 ± 4% to 67 ± 30% of PS1 (data not shown).

Senktide is a neurokinin-3 receptor preferring agonist [15] and was found to have no effect on the amplitude of PS1 or PS2. Using paired pulse stimulation with an interpulse interval of 20 ms, 10 μM senktide perfused for 10 min had no significant effect on the amplitude of PS2 (control amplitude of PS2 was 33 ± 8% of PS1 compared to 43 ± 5% of PS1 at the end of drug perfusion, not significant, n = 4, figure 3). In the same slices, after a 30-min washout, 8 μM substance P was perfused for 10 min and it again increased PS2 amplitude from 33 ± 7% of PS1 to 60 ± 5% of PS1 at the end of drug perfusion (p < 0.05, data not shown).

### Effect of neurokinin-1, neurokinin-2 and neurokinin-3 receptor antagonists on the action of substance P

To further characterise the receptor via which substance P decreased paired pulse depression, we next used three selective antagonists.

**Figure 4:**
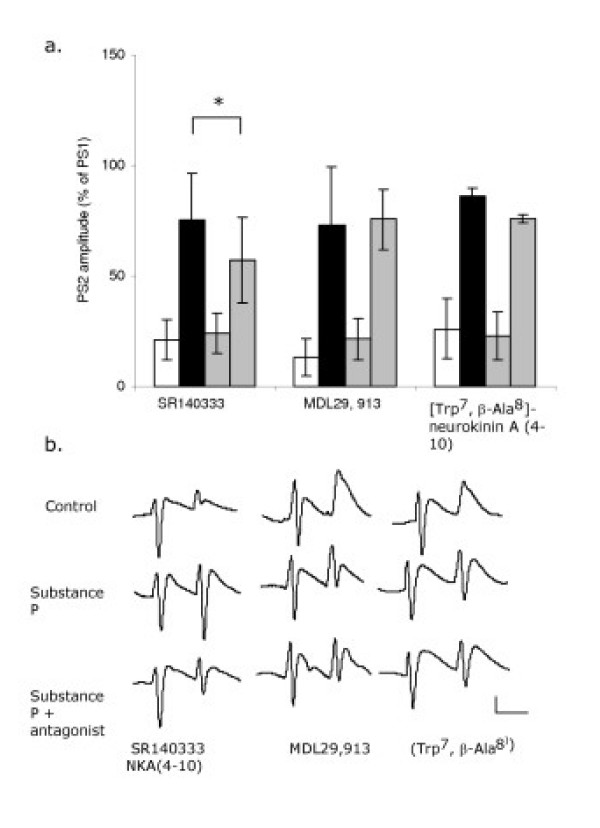
The effect of tachykinin receptor antagonists, when perfused with substance P. (a) Pooled data showing for each antagonist; control paired pulse depression using an interstimulus interval of 20 ms (PS2 amplitude expressed as a % of PS1, open bars), the effect of 8 μM substance P (filled bars), the effect of antagonist alone (diagonally hatched bars) and the effect of substance P in the presence of SR140333, MDL29,913 or [Trp^7^, β-Ala^8^]-neurokinin A (4–10) (horizontally hatched bars). The neurokinin-1 receptor antagonist SR140333 (12 μM) significantly reduced the effect of substance P. * denotes P > 0.05, n = 6. The neurokinin-2 receptor antagonist MDl29,913 (12 μM, n = 4) and the NK-3 antagonist [Trp^7^, β-Ala^8^]-neurokinin A (4–10) (5 μM, n = 3), had no significant effecton the action of substance P (b) Example synaptic response recorded from 3 representative slices showing control responses (top row), responses recorded in the presence of substance P (middle row), and responses recorded in the presence of substance P and the antagonist (bottom row). Scale bar represents 1 mV and 10 ms.

SR140333, a non-peptide neurokinin-1 preferring antagonist, was obtained from Sanofi research [16]. To establish a within-slice control, 8 μM substance P was first perfused for 10 min and, using an interpulse interval of 20 ms, this again caused an increase in the amplitude of PS2, as previously seen. After a 30-min washout period, during which PPD returned to control levels, 12 μM SR140333 was perfused for 20 min prior to, and during, a second perfusion of substance P. SR140333 had no effect on either PS1 or PS2 when perfused alone. However, when 8 μM substance P was perfused in the presence of SR140333, the effect of substance P on PS2 was reduced (figure 4). Substance P increased the amplitude of PS2 from 21 ± 8% of PS1 to 75 ± 19% of PS1, and when applied in the presence of SR140333 it increased PS2 from 19 ± 8% of PS1 to 54 ± 17% of PS1 (n = 6, p > 0.05).

MDL 29,913 is a neurokinin-2 receptor antagonist [17]. Using the same protocol as described above, the effect of 8 μM substance P was established and allowed to recover, before 5 μM MDL29,913 was applied for 20 min prior to, and during, a second perfusion of substance P. MDL 29,913 was found to have no effect itself on PS1 or PS2, or to block the effect of substance P. Substance P perfused for 10 min caused an increase in PS2 from a control amplitude of 13 ± 8 of PS1 to 73 ± 26 of PS1 at the end of drug perfusion. When applied in the presence of MDL 29,913, substance P still increased the amplitude of PS2 to 76 ± 13 of PS1 (n = 4, figure 4).

[Trp^7^, β-Ala^8^]-neurokinin A (4–10) is a neurokinin-3 receptor-preferring antagonist [18]. Using the same protocol as described above, 5 μM [Trp^7^, β-Ala^8^]-neurokinin A (4–10) was applied for 20 min prior to, and for 10 min during, application of substance P. [Trp^7^, β-Ala^8^]-neurokinin A (4–10) was found to have no effect itself on PS1 or PS2, or to block the effect of substance P. Within slice controls showed that 8 μM substance P perfused for 10 min caused an increase in PS2 from a control value of 26 ± 13% of PS1 to 86 ± 3% of PS1 at the end of drug perfusion (n = 3, figure 4). When 8 μM substance P was perfused in the presence of 5 μM [Trp^7^, β-Ala^8^]-neurokinin A (4–10), substance P increased the amplitude of PS2 to 76 ± 2% of PS1.

## Discussion

### Substance P selectively decreases paired pulse depression

The most striking feature of our results is the selective effect of substance P on PS2, but not PS1. Tachykinin receptors are located on inhibitory interneurones and not pyramidal cells [8] and neurokinin-1 receptors in particular are located on the cell body and dendrites of GABA immunopositive interneurones [3]. The location of the neurokinin-1 receptors would suggest an involvement of substance P in the control of inhibition of pyramidal neurones, and maybe of other interneurones, but not in directly modulating excitatory transmission. This is consistent with the fact that substance P had no effect on the recorded fEPSP or on PS1, which are primarily mediated by AMPA receptors. This result is, however, in disagreement with previous work. Kouznetsova and Nistri [9] found that perfusion of substance P (2–4 μM) and its synthetic analogue, substance P methyl-ester, significantly depressed field potentials recorded from the CA1 region of the mouse hippocampus. The reason for this discrepancy may be due to species difference (mouse vs. rat), or alternatively to the baseline recording conditions, and specifically the level of GABAergic inhibition. Kouznetsova and Nistri [9] hypothesised that substance P exerted its depressant action via GABA interneurones and not directly on the pyramidal cells recorded from. In our experiments, we deliberately selected hippocampal slices that exhibited good paired pulse depression, and therefore robust GABAergic inhibition. If this inhibition was effectively maximal, then substance P may be unable to further enhance it. It is therefore significant that we have previously noted paired pulse depression (and therefore presumably GABAergic transmission) is much weaker in slices maintained in a submersion chamber of the type used by Kouznetsova and Nistri, than an interface chamber as used in our experiments.

Since we could not demonstrate any effect of substance P on synaptic responses to single pulse stimulation, we turned to the phenomenon of paired pulse depression. Using an interstimulus interval of 20 ms, substance P (8 μM) perfused onto slices that displayed paired pulse depression, increased the amplitude of PS2 with no effect on PS1. Paired pulse depression of population spikes is thought to be predominately caused by feedback inhibition and can be used as an index of the strength of GABAergic neurotransmission within the hippocampus [12]. As previously noted, neurokinin-1 receptors are located on interneurones of the hippocampus, and substance P acting at these receptors could regulate the release of GABA. A decrease in GABA release would decrease the amplitude of the GABAergic IPSP evoked in the pyramidal neurone, increasing the probability that a second stimulus would fire an action potential, thereby increasing PS2 and inhibiting paired pulse depression. This effect was found to occur only at shorter interstimlus intervals of below 80 ms, which corresponds with the time course of the intracellularly recorded GABA_A _receptor mediated IPSP evoked in the CA1 pyramidal neurones [19]. An effect on inhibitory synaptic transmission is supported by the anatomical localisation of substance P receptors to GABA-containing interneurones and not to glutamate containing principal (pyramidal) cells [7]. Furthermore, electrophysiological recordings show that neurokinin-1 receptor agonists depolarise interneurones and increase the frequency of spontaneous (action potential dependent) inhibitory post synaptic currents (IPSCs) recorded from pyramidal cells in the CA1 region of the hippocampus [10]. Whilst these experiments showed an increase, rather than a decrease in the frequency of spontaneous IPSCs, they did not investigate the effect of substance P on evoked IPSCs.

### Effects of selective tachykinin receptor agonists and antagonists

A range of selective tachykinin receptor agonists was investigated. The neurokinin-1 receptor agonist substance P methyl ester was selected because it had been previously found to effectively mimic the effects of substance P in the mouse hippocampus, where it was effective in a concentration range of 10 nM–5 μM, with the maximum depressant action on field potentials observed using 0.1 μM [9]. In our experiments, perfusion of substance P methyl ester (0.5 μM), mimicked the effect of substance P and caused a significant increase in the amplitude of PS2. Due to the different concentrations used (8 μM and 0.5 μM), it is not possible to comment on the relative potencies of substance P and substance P methyl ester in our experiments. [β-Ala^8^]-neurokinin A (4–10) has been found to be a highly selective neurokinin-2 receptor agonist which has a 100-fold higher potency for neurokinin-2 receptors than for neurokinin-1 receptors [14]. 10 μM [β-Ala^8^]-neurokinin A (4–10) had a small effect on the amplitude of PS2 although this was not statistically significant. Any effect of [β-Ala^8^]-neurokinin A (4–10) on PS2 may be mediated via neurokinin-2 receptors, or more plausibly, [β-Ala^8^]-neurokinin A (4–10) may have some effect on neurokinin-1 receptors [20,21]. Such a weak interaction of neurokinin-2 receptor agonists with neurokinin-1 receptors has been reported in the entorhinal cortex, where [β-Ala^8^]-neurokinin A (4–10) mimicked the action of substance P in increasing GABA release from interneurones; an effect which was blocked by a neurokinin-1 receptor antagonist [10]. The neurokinin-3 receptor agonist senktide is one of the most potent of the neurokinin-3 receptor agonists [22]. Perfusion of 10 μM senktide had no effect on the amplitude of PS2, suggesting that the neurokinin-3 receptor is not involved in the decrease observed in paired pulse depression and has no effect on synaptic transmission measured here. A comparison of the effects of the agonists used gives an order of potency of substance P > [β-Ala^8^]-neurokinin A (4–10) > senktide. This is consistent with the effect of substance P on paired pulse depression being mediated by the neurokinin-1 receptor.

To further characterise the receptor involved, three tachykinin antagonist were used in an attempt to block the action of substance P. The selective neurokinin-1 receptor antagonist SR140333 (12 μM) significantly blocked the effect of substance P, although not completely. In the guinea pig ileum it was found that for SR140333 to have its full activity, a long contact time with the tissue was required [16] and this has been suggested to be longer than 120 min [23]. Contact time in the experiments performed here was a total of 30 min so an even better block may have been achieved with a longer perfusion time. Nevertheless, the ability of SR140333 to reduce the effect of substance P on paired pulse depression is consistent with the effect being neurokinin-1 receptor mediated. This is further supported with complete lack of effect of the neurokinin-2 receptor antagonist MDL29,913, and the neurokinin-3 receptor antagonist [Trp^7^, β-Ala^8^]-neurokinin A (4–10). An internal control was used in these experiments, which involved substance being applied twice to the same slice, firstly in the absence, and then in the presence of the antagonist. Under these conditions, desensitization of receptors might be expected to result in a smaller second response, independent of any antagonist effects. However, the fact that repeated perfusion of substance in the presence of the NK2 and NK3 antagonists produced substantially the same effect suggest that this is not the case, and that the reduced effect of substance P in the presence of SR140333 is due to antagonism of neurokinin-1 receptors.

The resulting order of potency of the agonists, and the effectiveness of the antagonists, therefore both suggest that the effect of substance P on paired pulse depression is mediated by neurokinin-1 receptors.

### Future work

Whilst the results of our experiments are consistent with the action of substance P being mediated by neurokinin-1 receptors, a more definitive proof of this would be to perform similar experiments in neurokinin-1 receptor knockout mice to establish whether substance P still inhibits paired pulse depression in these animals. There is some evidence that central tachykinin receptors may have different properties to the better characterised peripheral tachykinin receptors, and the possibility remains that the central effects of substance P are mediated by a distinct gene product, albeit with similar properties.

The effect of substance P in selectively decreasing paired pulse depression is consistent with a decrease in GABAergic inhibitory feedback inhibition of CA1 pyramidal cells, and such a mechanism is supported by the anatomical localisation of neurokinin-1 receptors in the hippocampus. However, other factors also contribute to paired pulse depression [24] and therefore, to investigate this hypothesis further, future experiments should use intracellular or whole-cell patch recordings from both pyramidal cells, and from interneurones in the CA1 region. Whilst there is convincing evidence that substance P inhibits spontaneous GABA release from interneurones [10], the effect of substance P on evoked IPSPs has not been determined. Such experiments will give further insights into the role of substance P in the central nervous system.

## Conclusion

The results show that perfusion substance P causes a selective reduction in paired pulse inhibition of population spikes evoked in the CA1 region of the rat hippocampal slice, and that this effect is mediated by NK1 receptors. This is consistent with the notion that NK1 receptors are present on the terminals of inhibitory interneurones and act to regulate GABA release.

## Methods

### Slice preparation

Young adult female Sprague Dawley rats (aged 4–6 weeks) were deeply anaesthetised using halothane and the brain removed and placed in chilled (4–5°C) oxygenated artificial cerebrospinal fluid (aCSF). The aCSF contained (in mM): NaCl 124, KCl 3, NaHCO_3 _26, NaH_2_PO_4 _1.25, D-glucose 10, MgSO_4 _1 and CaCl_2 _2 and was continuously bubbled with 95%O_2_/5% CO_2_. After dissecting free the hippocampus, 400 μm transverse slices were cut using a McIlwain tissue chopper. Slices were stored in a holding chamber at room temperature before being transferred to an interface type-recording chamber. Within the interface chamber, aCSF was continually perfused below the slice at a rate of 1–2 ml/min and at a constant temperature of 27–29°C.

### Extracellular recordings

Extracellular field recordings were obtained from the CA1 region using a glass recording electrode filled with 3 M NaCl. The recording electrode was placed in *stratum pyramidale *for recording population spikes, or in *stratum radiatum *for fEPSP measurements. Population spikes and fEPSPs were evoked by a bipolar silver stimulating electrode placed in *stratum radiatum *towards the CA3 end of the CA1 region to stimulate the Schaffer collateral commissural fibres. Constant current stimulus pulses of 0.02 msec width and 2–11 V were set to elicit a response of approximately half-maximal amplitude. Paired pulse stimulation at interpulse intervals between 20 and 150 ms were used in order to identify effects on both the first population spike (PS1), and the extent of paired pulse depression of the second population spike (PS2). Synaptic responses were evoked every 30 s and collected and analysed using the LTP program [25].

### Drugs and data analysis

All drugs were first dissolved in either water or dimethylsulphoxide (DSMO) according to the suppliers instructions (see below) to make a stock solution of at least 1000 times the final concentration. All stock solutions were kept frozen until needed. Application of drugs was achieved by dilution of stock solution into the aCSF, which was perfused onto the slice for the required time. Substance P and MDL 29,913 were obtained from Tocris (UK); substance P methyl-ester, [β-Ala^8^]-neurokinin A (4–10), senktide, spantide II and [Trp^7^, β-Ala^8^]-neurokinin A (4–10) were obtained from Bachem (UK); WIN 51708 was obtained from RBI Sigma (UK). SR140333 was a gift from Dr X. Emonds-Alt (Sanofi Research, Montpelier, France). Stock solutions of substance P, substance P methyl-ester, senktide and MDL 29,913 were all made up in water, whereas spantide II, [β-Ala^8^]-neurokinin A (4–10), [Trp^7^, β-Ala^8^]-neurokinin A (4–10), SR140333 and WIN 51708 were made up in DMSO. To facilitate pooling of data, fEPSP slopes or population spike amplitudes were normalised and expressed as a percentage of the mean slope or amplitude recorded during the entire 15 min control period before addition of drugs. In experiments using paired pulse stimulation, the extent of paired pulse depression was determined by expressing the amplitude of PS1 as a percentage of the amplitude of PS2. All graphs represent pooled data from 3–9 slices prepared from different animals and plot the mean ± standard error of the mean (s.e.m.). Statistical analysis of the raw (ie not normalised) data involved the use of a Students paired t-test to compare control fEPSP slope, population spike amplitude, or extent of paired pulse depression with that recorded at the end of drug perfusion. The control response was measured from the average of the last 5 consecutive responses before drug perfusion, and the drug response was measured from the average of the last 5 consecutive responses during drug perfusion. p < 0.05 was taken as significant.

## Authors' contributions

KNW carried out all the experiments and performed the analysis of the data. SND conceived of the study and participated in its design and coordination. KNW and SND drafted the manuscript. All authors read and approved the final manuscript.
